# Abiotic and biotic factors associated with the presence of *Anopheles arabiensis* immatures and their abundance in naturally occurring and man-made aquatic habitats

**DOI:** 10.1186/1756-3305-5-96

**Published:** 2012-05-19

**Authors:** Louis Clément Gouagna, Manpionona Rakotondranary, Sebastien Boyer, Guy Lempérière, Jean-Sébastien Dehecq, Didier Fontenille

**Affiliations:** 1 Institut de Recherche pour le Développement (IRD), UM1-CNRS 5290-IRD 224: Maladies Infectieuses et Vecteurs – Ecologie- Génétique, Evolution et Contrôle (MIVEGEC), Montpellier, France; 2 Centre de Recherche et de Veille sur les maladies Emergentes dans l’Océan Indien (CRVOI) Sainte Clotilde, Reunion Island, France; 3 Service de lutte anti vectorielle, Agence Régionale de Santé (ARS) Océan Indien, Saint-Denis, Reunion Island, France

## Abstract

**Background:**

*Anopheles arabiensis* (Diptera: *Culicidae*) is a potential malaria vector commonly present at low altitudes in remote areas in Reunion Island. Little attention has been paid to the environmental conditions driving larval development and abundance patterns in potential habitats. Two field surveys were designed to determine whether factors that discriminate between aquatic habitats with and without *An. arabiensis* larvae also drive larval abundance, comparatively in man-made and naturally occurring habitats.

**Methods:**

In an initial preliminary survey, a representative sample of aquatic habitats that would be amenable to an intensive long-term study were selected and divided into positive and negative sites based on the presence or absence of *Anopheles arabiensis* larvae. Subsequently, a second survey was prompted to gain a better understanding of biotic and abiotic drivers of larval abundance, comparatively in man-made and naturally occurring habitats in the two studied locations. In both surveys, weekly sampling was performed to record mosquito species composition and larval density within individual habitats, as well as *in situ* biological characteristics and physico-chemical properties.

**Results:**

Whilst virtually any stagnant water body could be a potential breeding ground for *An. arabiensis*, habitats occupied by their immatures had different structural and biological characteristics when compared to those where larvae were absent. Larval occurrence seemed to be influenced by flow velocity, macrofauna diversity and predation pressure. Interestingly, the relative abundance of larvae in man-made habitats (average: 0.55 larvae per dip, 95%CI [0.3–0.7]) was significantly lower than that recorded in naturally occurring ones (0.74, 95%CI [0.5–0.8]). Such differences may be accounted for in part by varying pressures that could be linked to a specific habitat.

**Conclusions:**

If the larval ecology of *An. arabiensis* is in general very complex and factors affecting breeding site productivity sometimes not easy to highlight, our results, however, highlight lower populations of *An. arabiensis* immatures compared to those reported in comparable studies conducted in the African continent. Overall, this low larval abundance, resulting from both abiotic and biotic factors, suggests that vector control measures targeting larval habitats are likely to be successful in Reunion, but these could be better implemented by taking environmental variability into account.

## Background

Tropical areas are ideal zones for mosquito-transmitted diseases. The majority of these diseases are caused by protozoan parasitosis, filariasis and arboviruses, which constitute serious public health risks in developing countries. In tropical areas worldwide, these diseases remain among the most significant to human health, due to considerable rates of morbidity and mortality. The most largely widespread is malaria, for which 216 million cases and 655,000 deaths are recorded each year according to a recent census [1], with 81% of cases and 91% of deaths estimated to occur in Saharan Africa. In geographic areas where malaria has been eradicated or at least controlled to a certain extent, sporadic epidemics can sometimes occur, or re-emergence may eventually cause significant recrudescence. On Reunion island (21°.12″S, 55°.5″E), for example [2], malaria was eradicated in the 1970s by large scale spraying campaigns of chemical pesticides (including DDT and temephos) and by the mass use of antimalarial drugs [3,4]. Nowadays, the Regional Health Agency (ARS) estimates that approximately 113 cases of malaria are imported to Reunion from the neighbouring islands every year [5]. The presence of *Anopheles* mosquitoes, capable of transmitting the disease [6], and the increasingly frequent record of these imported malaria cases [5,7], together suggest a real threat of re-emergence of malaria and a frightening public health challenge in terms of disease prevention.

Reunion Island is home to 12 mosquito species [6], among which is, *An. arabiensis*, which is a sibling species of *An. gambiae*. The exact origin of this vector on Reunion Island is unknown, but the same species is abundant on many neighbouring islands, such as Madagascar and the Comoros [6,8], and also in several countries in the South-eastern coast of Africa [9,10]. *An. arabiensis* is currently the only species of the complex *Anopheles gambiae* on the island [10,11]. *An. arabiensis* demonstrates a preferred exophilic and exophagic lifestyle [12,13], and therefore a larval control program, consisting of regular application of *Bacillus thuringiensis* serovar *israelensis,* ranks among the top priorities for the island’s public health management, aiming both at decreasing the presence of vectors and reducing the risk related to the diseases that they could transmit. In spite of regular treatment in well identified and accessible aquatic breeding habitats, their distribution is spreading in certain inhabited zones. The general geographic distribution of *An. arabiensis* breeding habitats is being extensively monitored and mapped by local health authorities. A recent analysis of a 14 years dataset from larval surveys suggested that whereas this mosquito species was formerly present on almost the entire island, there is now evidence of discontinuity in the range distribution of suitable habitats [14]. This dates from the period that followed the large control campaigns against *An. arabiensis* predominantly in urbanized areas [3,4,13]. In rural areas, man-made larval habitats are by far the most important, but like the natural ones, they depend primarily on rainwater for their persistence.

Our knowledge of the distribution of *An. arabiensis* in Reunion is based mainly on broad spatial and temporal averages of breeding site occurrence in a wide range of habitat types [14], and therefore does not accurately represent the conditions and processes driving larval abundance patterns in potential habitats. An account of previous studies, primarily in sub-Saharan Africa, indicates that several environmental factors determine larval density and may influence the development/survival rate of the malaria vector larvae [15-20]. These factors include climate, physical and chemical conditions of the aquatic habitats, land cover and vegetation type, and biological characteristics. No similar study has been purposely setup to shed light on the environmental factors that are associated with the productivity of *An. arabiensis* breeding sites in an island context such as in Reunion. Here, cross sectional surveys were undertaken both to determine which factors are important to discriminate among aquatic habitats with and without *An. arabiensis* larvae, and to understand biotic and abiotic drivers of larval abundance, comparatively in man-made paddle pools and naturally occurring rock pools. Information on measurable changes in the abundance of larvae can be used to determine how environmental factors and control measures are influencing population persistence in these potential habitats.

## Methods

### Study areas

**Figure 1:**
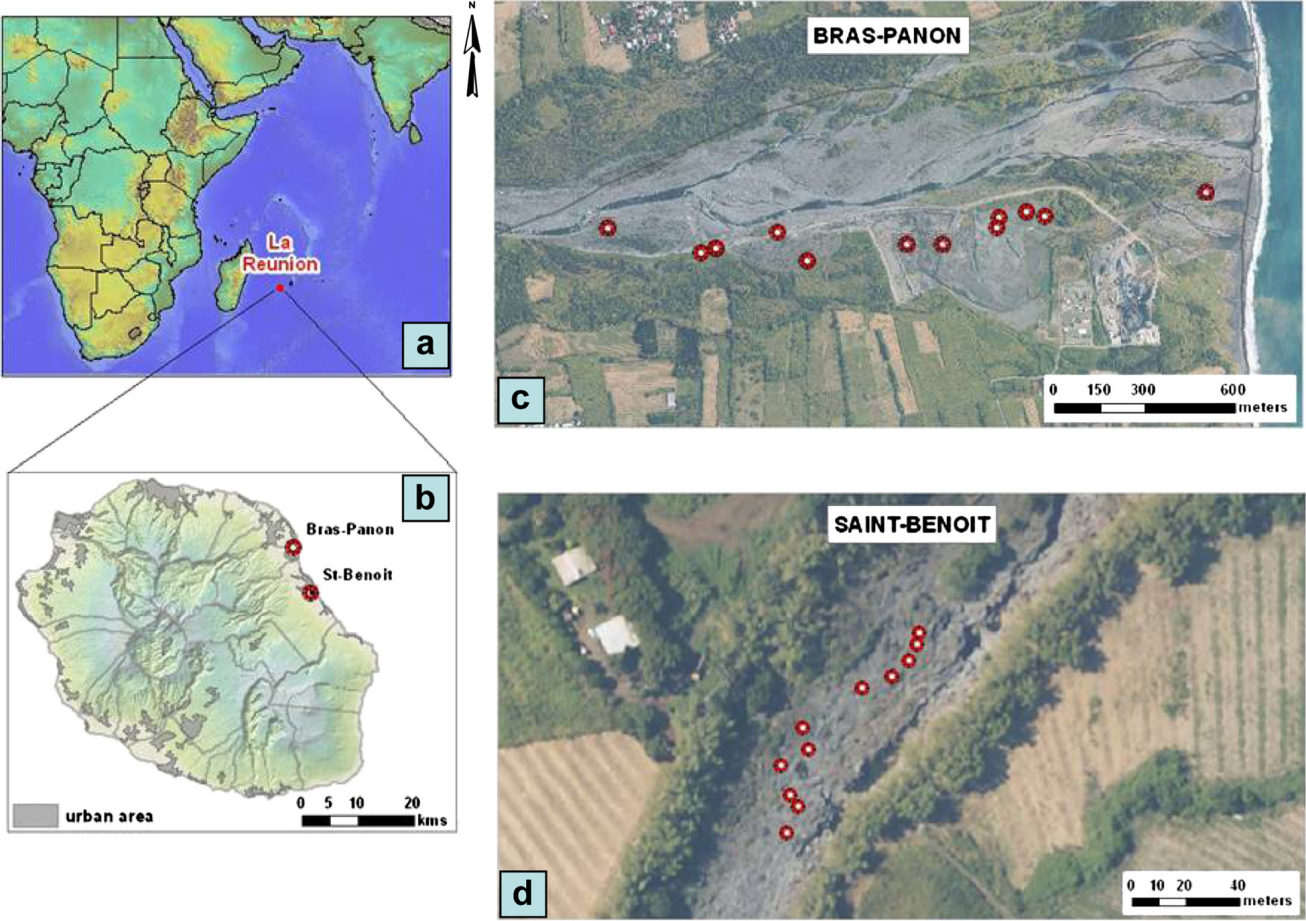
Map showing the topography of the study areas and locations of sampled larval habitats.

Two larval surveys were undertaken from January to February 2010 and from January to April 2011 in Bras-Panon (20°59′ 5.72″ S; 55°41′ 12.14″ E) and Saint Benoit (21°1′ 60″ S, 55° 43′ 0″E), two spatially distinct zones that encompass 88.5 km² and 229.6 km², respectively (Figure 1a–b). These studied zones are 15 km apart and are located 20–35 km northeast of the capital Saint Denis.

The first larval inspection survey was conducted in January-February 2010 in Bras-Panon. This open area (situated at 700–822 meters above sea level) is characterized by a large stone quarry (Figure 1c). Vast areas in stone pits dug to about 1–10 meters in depth and excavated earth are regularly filled by precipitation and infiltration from the Mat River that skirts the stone quarry. The water stream runoff and the retreat of a rain torrent, which occasionally flood greater areas, leave numerous pools as potential breeding habitats for mosquitoes*.* This study area is known to host only *An. arabiensis* species [11-14]. Located in the South West of The Indian Ocean, the geographic isolation of this subtropical island makes natural migrations rare [10], and according to ongoing inventories no other *An. gambiae* sibling than *An. arabiensis* have been recorded in recent years.

Following the preliminary survey described above, a second survey was prompted in January-April 2011 to gain a better understanding of biotic and abiotic drivers of larval abundance. In addition to the same area mentioned above, a second location in Saint Benoit was included for comparison purposes. One particular topographic feature that characterises this site is the existence of a ravine (475 meters in length) with stones along its contour approximately 4 m in depth by 6 m in width (Figure 1d). Often rock pools occurring in exposed areas of the bedrock may store water for prolonged periods of time – usually from November to April - providing a source for mosquito proliferation

**Figure 2:**
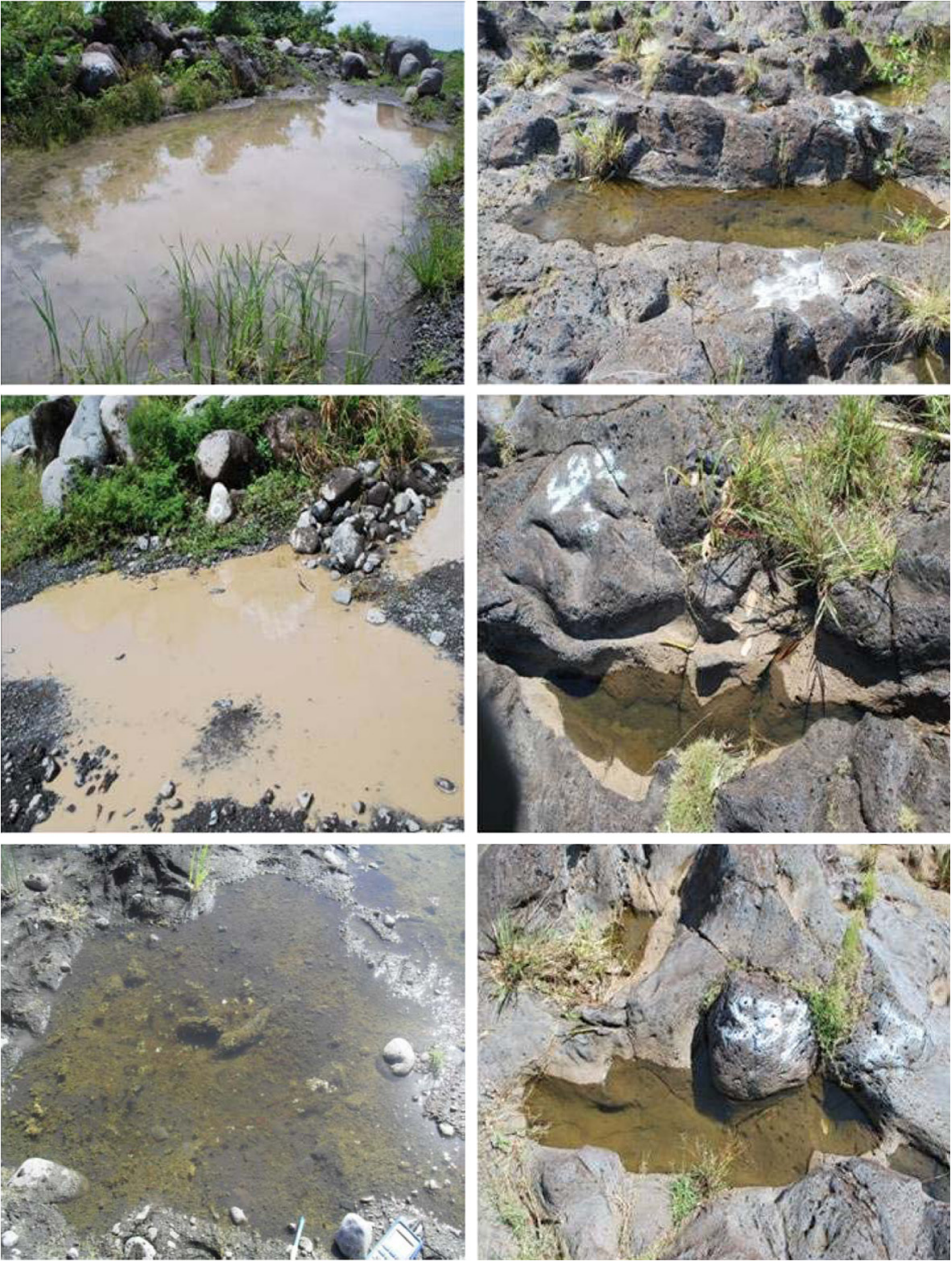
Typical aquatic habitats sampled-Man-made (left column) and naturally occuring rock pools (right column).

At points both along the stone quarry in Bras-Panon and along the ravine in Saint-Benoît (Figure 1c,d), twelve aquatic habitats that would be amenable to an intensive long-term study, and which were representative of *Anopheles* breeding sites of each type were selected. The selection of positive habitats was based on the presence of larvae (by dipping method) and the selection of negative habitats was also made among the prevailing aquatic habitats in the studied locations (Figure 2). Painted numbers with white paint identified the selected habitat when visual tracing would not be evident. Marking was also to minimize disturbance. The precise co-ordinates of these selected breeding sites, separated from each other by 5–10 meters, were also recorded by GPS (Garmin inc., GPSmap 60CSx). Field visits were carried out on a weekly basis, usually from 8 to 11 a.m. at the pre-selected aquatic habitats.

### Abiotic factors associated with the presence of an. Arabiensis breeding sites

Initial visual inspection was performed in Bras Panon from January to February 2010 on a daily basis, to ensure identification of every depression filled with water during the season. Subsequently, a representative sample of 28 discrete aquatic habitats, at distances ≥30 m from each other, were selected and categorized into habitats with (positive) and without (negative) *Anopheles* larvae. For 2 consecutive months, weekly visits were undertaken during which 5–10 dips were taken from each aquatic habitat to confirm the presence or absence of mosquito larvae. An aquatic habitat was classified as positive when at least one anopheline larva was present in the sample. Water current was determined by measuring the speed of drainage of a piece of paper as a function of time (in seconds) and a distance (given in centimeters). The water depth was measured in 3 various points of each pool using a wooden meter ruler inserted in the water until it touched the solid bottom. Habitat length and width (cm), water depth (cm), and water surface area were calculated in square metres. In addition, water current measurements of conductivity were taken at the same time as those of pH and water temperature using two thermo-pH-meters (Hanna Instruments, Lingolsheim, France) that were plunged 3 times under the waters’ surface in 3 distinct points.

### Biotic factors associated with the presence of an. Arabiensis breeding sites

An area sampler was used to improve the detection of potential microorganisms present in individual breeding habitats [21,22]. We used a bottomless plastic tray to delimit a sampling quadrat of 1750 cm² (length: 50 cm, width: 35 cm, height: 28 cm). Depending on the size of the habitats, 2–3 quadrats were examined for the number of species of macro-organisms (including *An. arabiensis*) present. In the positive breeding sites, samples of mosquito larvae were recovered by means of a standard pint dipper (Bioquip, Gardena, CA, USA), in addition to net collections [23]. As diver species may remain at the muddy bottom of the habitat, we further excavated the bottom of each habitat to detect potential micro invertebrates. A total of 15 minutes sorting was completed for each water body and for small habitats the inventory stopped when all of the species of macrofauna present in the sampling quadrats were collected.

Mosquito larvae and cohabiting fauna scored were returned to the respective habitat after sampling. Given the small sample of larvae observed in positive breeding sites (range 0–1 larvae per 5 dips) no attempt was made to quantify larval productivity. However, the Shannon diversity index (H’), which takes into account the relative abundance of species *i* (*Ni*) relative to the total number of species present (*N*) [24], was calculated for each habitat. Vegetation type and grass or algal cover rate in relation to the total surface area of the aquatic habitat were also determined. Algae or vegetation cover was further derived from a ratio of total area covered with vegetation or algae over the estimated surface of the corresponding breeding site. This was classified as one of the following five groups: zero if vegetation or algae were not represented in any habitat sampled, 1: ≤ 24% of surface coverage, 2: 25–49%, 3: 50–74% and 4: 75–100%. Photographs of each habitat were taken to confirm these estimates. The dominant vegetations consisting of plant parts or plant flowers were also collected from each aquatic habitat, and then preserved in newspaper for later identification in the laboratory.

### Estimation of larval productivity in relation to in situ biotic and abiotic factors

During the second survey at preselected aquatic habitats, weekly sampling was performed to record species composition, density of larvae and other physical and biological characteristics of the habitats. For any breeding habitat, several dips were made at equal intervals around the habitat’s edges using a standard dipper and examined for the presence or absence of mosquito larvae. The number of dips was dependent upon the size of the habitats and water level (2 dips: < 1 m; 4 dips: 1 m ≤ perimeter < 2 m; 6 dips: 2 m ≤ perimeter < 5 m; 10 dips: 5 m ≤ perimeter < 10 m; 12 dips: >10 m). Contents collected in the dip were emptied onto a white enamel tray to facilitate counting of larvae and cohabiting microorganisms. All mosquito larvae and associated aquatic organisms were left *in situ* whenever possible, with the exception of a few occasions, when voucher samples were collected for identification in the laboratory. For mosquitoes, species-specific polymerase chain reaction (PCR) was implemented to confirm the results of initial morphologic identifications.

### Climatic variables

The two study sites receive heavy precipitation and the temperature and humidity conditions show sharp diurnal and seasonal fluctuations [14]. The wet season (October to April) is warmer and more humid. Although occasional *Anopheles* breeding is known to occur during other months of the year, evidence derived from previous studies indicates that the period from November to April encompasses the majority of breeding events [11,14]. During this study, the rainfall records and the measurements of temperature and relative humidity were taken into account in order to better describe the climate within each studied site. The local meteorological stations in Bras-Panon (BP) and Saint Benoit (SB) provided weekly climatic data for 3 consecutive months, covering the same interval as the larval abundance dataset.

### Data analysis

The initial preliminary survey had divided aquatic habitats into positive and negative sites based on the presence or absence of *Anopheles arabiensis* larvae. Pearson’s chi-square test and the non-parametric Wilcoxon test were applied to analyze statistical differences in ecological parameters among the habitat categories. The association between presence or absence of *An. arabiensis* larvae and environmental parameters was tested by logistic regression. Additional statistics used the Principle Component Analysis (PCA) to detect characteristics that best discriminate the negative and positive habitats. This analysis was made separately for biotic (micro-fauna and flora) and abiotic (water surface, flow velocity, temperature, pH and turbidity) factors as scored within the positive and negative habitats.

In the second survey, we used the species-specific measure of average larvae per dip summed across habitat type for each week as the response variable for the univariate General Linear Models (GLM). Unless stated otherwise, all aquatic larval stages were combined into one measure of species abundance (average number of larvae per dip) within a given habitat. Continuous measures (temperature, pH, surface, depth) were log transformed and proportions (habitat coverage with filamentous algae, emergent plants) were arcsine transformed to normalize the data before the analyses. Study area and habitat identity were included as independent fixed factors, week number was considered as the within subject variable, water depth and surface area, water temperature, conductivity and pH as covariates, whereas variables in the following list were considered as random factors: algae and vegetation type, algae and vegetation cover and macro-fauna species composition. All non-significant terms were sequentially dropped to yield a minimum model, which took into account only factors found to significantly affect the presence and abundance of mosquitoes in habitats. Pairwise comparison of mosquito productivity with habitat type, and week was done using Tukey’s HSD test of GLM with repeated measures. The Shannon diversity index was calculated for the floro-faunistic components of each habitat. The SPSS statistical package (SPSS Inc) version 18.0 for Windows was employed for the analyses. Means and standard errors are reported throughout; all tests were two-tailed, and significance was assigned at the 5% level.

## Results

### Description of An. arabiensis positive and negative aquatic habitats

**Figure 3:**
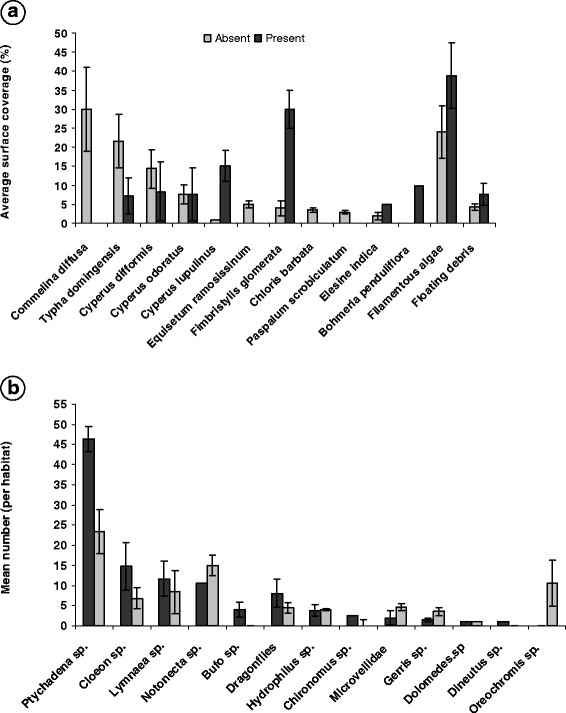
**Composition of plant species (a) and aquatic invertebrate orders b) associated with aquatic habitats with and without** ***An. arabiensis*** **larvae in different in Northeast of La Reunion Island.**

Preliminary study on the measurable environmental characteristics linked with the presence and absence of *A. arabiensis* larvae was performed each week in a total of 28 distinct aquatic habitats (14 per habitat type). *An. arabiensis* was the only mosquito species present in positive habitats during the sampling period. Without exception, all *Anopheles* positive habitats had usually very low numbers of immatures (usually one larva in a total of 5–10 dips) on successive sampling weeks. This scarcity of mosquito larvae made trends in larval abundance exceedingly difficult to detect reliably. Table 1 summarises the general characteristics of aquatic habitats sampled with and without *Anopheles* larvae and additional site specific features are given in Figure 3 (a -flora, b-fauna). On average, the positive and negative habitats were not significantly different on the basis of size (Table 1) (F_1, 26_ = 3.5 *p* = 0.59). The mean water depths, vegetation cover, water temperature at sampling time, pH values, were also similar in the two biotopes. The interaction of the pH and turbidity showed a negative significant effect on the occurrence of *An. arabiensis* larvae within a given habitat (Wald *χ*^2^ = 7.79, df = 1, *p* = 0.004), whereas only turbidity, taken individually, seemed to distinguish the two aquatic biotopes.

**Table 1:** **Comparison of different group means (+ SEM) of environmental variables between aquatic habitats with and without** ***Anopheles arabiensis*** **larvae in Bras-Panon**

	Mosquito larvae		
Environmental variables	Present	Absent	*p*-value
Total number of habitats sampled	14	14	−
Flow (velocity in m/s))	Stagnant (0)	0.41 ± 1.4	=
Water body area (m2)	8.42 ± 7.2	5.81 ± 3.7	0.58**
Water depth (cm)	3.25 ± 1.1	3.51 ± 0.9	0.24**
Turbid/clear (%)	40%/60%	99.4%/0.6%	0.002*
Temperature (°C)	32.12 ± 3.9	31.93 ± 1.9	0.15**
pH	8.78 ± 1.26	8.61 ± 1.07	0.29**
% emergent vegetation	30%	45%	0.43*
% algae	45%	75%	0.05*
Number of macro-invertebrate species (diversity index)	12 (1.78)	10 (1.92)	0.014***
Number of grass species (diversity index)	11 (1.61)	9 (1.84)	0.001***

=: 30% of aquatic without *An. arabiensis* were slow moving while the remaining and those with larvae were stagnant. * Wald chi square test. ** GLM F-test. *** Wilcoxon test.

‘Positive habitat’ was defined as a water body which could contain at least one larva on any sampling visit, in contrast to ‘negative habitat’ which refers to habitat with no single larvae sighted from at least 5–10 dips (depending on size) on each occasion. These were selected among the commonly encountered aquatic habitats that were likely to hold water throughout the study period. The selection of aquatic habitats for sampling was done in a way as to reflect the diversity of water bodies present in individual study sites.

Emergent and floating vegetations and small clumps of filamentous algae were frequently observed in the majority (70%) of the aquatic habitats examined, with only a mean of 30–45% of the habitat surface covered (Table 1). One important observation is that *An. arabiensis* will not occupy all available aquatic habitats at any given point in time, and that habitat occupancy depends greatly on the plant species composition and on degree of habitat cover. Consequently, some plant species that occur in negative habitats were always absent in positive habitats and vice versa (Figure 3a). Overall, the floral diversity index was 4.6 for positive habitats against 4.0 for the negative ones. The Principal Component Analysis (PCA) on the flora selected 29% of the variables: 14% on the 1^st^ component-axis and 15% on the 2^nd^ axis.

Across the surveyed habitats, both species richness and the densities of multiple macro-invertebrate species (*Anopheles arabiensis* not included) at the surveyed sites differed between habitats occupied by *Anopheles* larvae and those where they were absent (Figure 3b). Although these contrasts were not necessarily consistent among the surveyed week, it was shown that dragonfly larvae (*Libellulidae: Diplacodes lefebvrii; Anax imperator; Orthetrum sp.; Ischnura senegalensis*) and *Ptychadena mascareniensis* and two fish species (*Oreochromis sp*. (*Tilapia*) and *Poecilia sp. (Guppy,* sighted but not captured) were most abundant in habitats where *An. arabiensis* larvae were absent, suggesting that the presence of these predators probably reduced the probability of habitat colonization by *An. arabiensis*.

The species composition, hence the total number of species at a given habitat was noted to be an increasing function of habitat size (species number = 1.653 ± 0.819 habitat size). The positive breeding habitats had a slightly lower diversity index (2.53) than that of the negative ones (2.83), but the difference was not statistically significant (F test, *p* = 0.82). PCA with regard to fauna detected 47% of the variables that best discriminate positive from negative habitats, with dragonflies, *Lymneae sp.* (Mollusc), V*elis sp. (Mesoveliidae)*, *Ptychadena mascareniensis*, and *Oreochromis sp* being the influential parameters. However, using the presence and absence of larvae as grouping variable, and mean density of each of these organisms as covariate variables, logistic regression models indicated that larger mean numbers of each species were not indicative of the distribution of the negative or positive aquatic bodies (Wald chi square, p > 0.05 in all cases)

### Abiotic factors associated with man-made and naturally occurring habitats

At the beginning of the second survey, just over 35.4% (17/82) of the man-made aquatic habitats and 41.1% (23/105) of the naturally formed rock pools were positive for anopheline immature stages, respectively in the stone quarry in BP and at the ravine of SB. The survey was carried out from the beginning of January to late April when average weekly rainfall ranged from 0.62–34.4 mm, with BP having more downpour on every sampled week than SB (site x visit, *F*_10, 107_ = 32*10^5^, *p* < 0.001). On average, the average daily air temperatures ranged from 21.8 to 32.2°C at BP and 20.9 to 33.6°C in SB. Limiting analyses to the aquatic bodies designated as positive for mosquito larvae (man-made habitats: 12, natural habitats: 11), all were exposed to direct sunlight and diverse in size. The average surfaces (*F*_1, 157_ = 40.9, *p* < 0.001), pH (*F*_1, 157_ = 31.03, *p* < 0.001) and conductivity (*F*_1, 157_ = 365.02, *p* < 0.001), recorded for man-made paddle pools were significantly greater than those of most of rock pools (Table 2). Water depths were greater in the latter than in the former habitat type (*F*_1, 157_ = 78.7, *p* < 0.001). At least once during the study period, 31% of the man-made habitats against 7% rock pools were slightly clear to turbid (Table 2). In parallel, water temperatures during the study period oscillated between 29°C and 33.23°C in man-made habitats against 29°C and 32.22°C in rock pools (*F*_1, 157_ = 5.19, *p* = 0.02). Mean temperature of the prospected breeding water bodies was negatively related to habitat mean water depths (Pearson’s correlation: r = −0.35, *p* < 0.001), but significantly increased with increasing pH (r = 0.4, *p* < 0.001) and conductivity (r = 0.26, *p* = 0.001).

**Table 2:** **Summary of major abiotic and biotic characteristics associated with two different type of aquatic habitats with** ***An. arabiensis*** **immatures in Bras-Panon and Saint-Benoît, North-east of La Reunion**

	Bras-Panon	Saint Benoît	*p*-value
Type of breeding site	Man-made	Rock pools	
Number of habitats	12	11	=
Total number of samples taken	82	105	0.002**
**Abiotic factors**			
Mean surface area (m2)	8.8 [5.04–12.5]	3.6 [2.5–4.6]	< 0.001**
Depth (cm)	7.1 [6.3–7.8]	12.1 [11.2–12.9]	< 0.001**
Temperature (°C)	33.2 [33.1–34.3]	32.2 [31.8–33.0]	0.02**
pH	8.5 [8.4–8.7]	7.8 [7.6–8.0]	< 0.001**
Conductivity (μS/cm)	206.3 [185.5–227]	28.9 [26.4–31.4]	< 0.001**
Turbidity	31%	7%	
**Biotic characteristics**			
Mosquito species detected	*An arabiensis*	*An. arabiensis, Culex neavei*	N/a
Total number of larvae	320	432	
Mean larval density per dip	0.55 [0.37–0.73]	0.70 [0.57–0.83]	0.03**
Macro-fauna species (diversity index)	14 (1.9)	15 (1.8)	0.45**
**Algae** (% occurrence)			
Absent	29%	62%	−
Low	48%	32%	−
Medium	21%	5%	−
Average surface coverage (%)	12.5%	6%	0.001*
**Floating and emergent vegetation**			
Absent	62%	57%	−
Low	32%	43%	−
Medium	6%	−	−
Average surface coverage (%)	9%	3%	0.031*

Mean and [95% Confident interval] for each parameter are provided.

* Pearson’s chi square test. ** GLM F-test with all analyses performed after log10 transformation.

### Biotic factors associated with man-made and naturally occurring habitats

**Figure 4:**
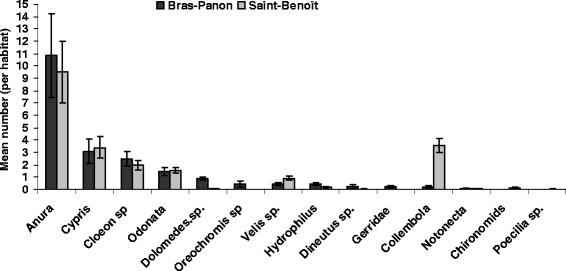
**Diversity and relative abundance of macro-invertebrates recorded in individual man-made and natural habitats occupied by *An. arabiensis*** **in distinct study locations.** * Note:* Man-made habitats were represented by standing water bodies within excavated soil dug by trucks or in wheel tracks at the stone quarry. Mainly rock pools represented natural habitat.

The most important observation was in relation to a difference in vegetation cover between the two habitats types. Indeed, margins of all breeding sites in BP had vegetation and submerged grass, e.g. *Typha domingensis* (Typhaceae) and 3 species of *Cyperacea* (*Cypesrus haspan*, *Cyperus difformis*, *Fymbristilis glomerata*) and green algae. At SB, the most represented grass species was *Cynodon dactilon* (Graminae: *Poaceae*). Differences in algal cover (*χ*^2^ = 22.8, df = 2, *p* < 0.001) and grass cover (*χ*^2^ = 39.1, df = 3, *p* < 0.001) rates between two sites were statistically significant. Consistent with the preliminary survey in BP, the main variables associated with the presence of *An. arabiensis* larvae in habitats were green algae and *Cyperaceae* plant family. *An. arabiensis* larvae were also found associated with approximately 13 macro-invertebrate species (Figure 4). More macro-invertebrates were likely to be sighted in man-made breeding habitats at BP and in naturally occurring rock pools at SB (Odd ratio = 1.38, 95% CI [1.01–1.9], *p* = 0.03). Both in the former habitat type (Wald *χ*^2^ = 42.2, df = 11, *p* < 0.001) and in the latter (*χ*^2^ = 33.5, df = 10, *p* < 0.001), the specific richness of macro-invertebrates varies greatly from one habitat to another as well as in time for man-made habitats (Wald *χ*^2^ = 7.7 df = 22, *p* = 0.005), but not for rock pools (*χ*^2^ = 42.21, df = 21, *p* < 0.00). The small size of rock pools made fish presence unlikely, but the *Poecilia reticulata* (*Cyprin odontiform*) and *Culex neavei* (Diptera) were the most represented species.

### Mosquito species composition and abundance of *Anopheles arabiensis* immature stages

Consistently at the two study sites, the number of larvae per dip was relatively low throughout the sampling period. Overall, 41 pupae and 1121 larvae (pooled data for all instars) were collected, including 369 *Culex neavei* and 432 *An. arabiensis* larvae recovered only at Saint Benoît and 320 larvae of *An. arabiensis* obtained at Bras-Panon. At the former site, 26% of the rock pools consistently supported both *An. arabiensis* and *Culex neavei* larvae. Within those, the average numbers of *An. arabiensis* and *Culex neavei* larvae per dip was not significantly different (F-test: *p* = 0*.*32). Only the data on *An. arabiensis* larval densities are presented, while their associations with *Culex* larvae or other factors related to the habitats are examined statistically (Table 2). The relative number of *An. arabiensis* larvae produced by individual breeding site was significantly greater in rock pools (mean ± SEM: 0.70 ± 0.6 larvae/dip) than in man-made habitats (0.55 ± 0.8 larvae/dip) (*F*_1, 185_ = 4.3, *p* = 0.03). The mean larval density per habitat fluctuated significantly from one visit to another (*F*_11, 164_ = 2.5, *p* = 0.005) and at each studied location (location x visit interaction: (*F*_10, 164_ = 2.56, *p* = 0.01) suggesting regular oviposition activity.

### Relationships between larval abundance and in situ biotic and abiotic factors

Considering the environmental variables with significantly different group means for sites with and without larvae, *An. arabiensis* larval density observed in rock pools correlated negatively with the water depth and rainfall and positively correlated with conductivity (Table 3). In man-made habitats on the other hand, the larval density recorded within the habitats was correlated with none of the physicochemical parameters. Considering the floral component of the habitat characteristics, we did not find a significant relationship between *An. arabiensis* larval densities and the various vegetation cover levels, either with algae or with emergent vegetation. Concerning the fauna component, the average larval density was positively correlated with the mean number of *Cypris sp* (*Ostracode*) as well in BP (F_1.79_ = 0.21; *p* = 0.014) as in SB (F_1.102_ = 4.50; *p* = 0.03). As the density of *Cypris sp* increased, breeding sites were observed to display the most intense larval activities. In addition and only in SB, a weak positive correlation was observed between the *An. arabiensis* larval density and the presence of *Hydrophilus sp*. (*Hydrophylidae*) (F_1.101_ = 6.68; *p* = 0.01)*.* Further analyses revealed that the relative abundance of *Culex neavei* larvae, but not anopheline larvae, showed a negative correlation with dragonflies (F_1.103_ = 4.51; *p* = 0.03) (Table 3).

**Table 3:** **Results of test statistics showing relationships between** ***Anopheles arabiensis*** **and** ***Culex neavei*** **larval densities and key environmental and physicochemical parameters**

Dependent variable	Site	Explanatory Variables	Correlation	Constant	*p*-value
*An. arabiensis* larval density	BP	Cypris sp.	0.10	0.131	0.014
	SB	Water depth	−0.25	0.30	0.007
		Conductivity	0.14	0.31	0.011
		Rainfall	−0.04	0.30	0.019
		Cypris sp.	0.09	0.16	0.005
		Hydrophilus sp.	0.22	0.16	0.036
*Culex neavei* larval density	SB	Water depth	−3.91	5.00	0.001
		*Dragonflies*	*−1.38*	*1.15*	*0.036*

*BP* Bras Panon, *SB* Saint Benoît, All variables included in the GLMs were considered on log-scale (Log10 [n +1]).

## Discussion

The present study was undertaken in an attempt to establish factors associated with aquatic habitat that may influence both the presence of *An. arabiensis* immatures and their densities. At the studied locations, Bras-Panon and Saint-Benoit, environmental features are eminently complex and may conceal a great number of factors which can interfere with larval development. Contrary to BP where only *Anopheles arabiensis* larvae could be found in distinct man-made paddle pools, naturally occurring rock pools at SB were characterized by the presence of *Anopheles arabiensis* and *Culex neavei*. The particular accent we put on *Anopheles arabiensis* stems from the interest in this species as a main target of the antivectorial control in Reunion Island. The results about the natural variability of larval abundance in relation with abiotic and biotic environmental factors were in concordance with preliminary observations concerning environmental variables that best predict the presence or absence of larvae in different habitats.

Whilst virtually any stagnant water body could be a potential breeding ground for *An. arabiensis*, we showed that standing water bodies occupied by *An. arabiensis* larvae had different structural characteristics when compared to those where larvae were absent. Our results indicate that the presence of immatures or their absence in some aquatic habitat was unrelated to parameters such as the surface, depth, temperature, pH, turbidity. On the other hand, the range of habitats where *An. arabiensis* larvae were absent were characterised by the highest frequency of sighting of predator fauna such as Tilapia fish (*Oreochromis sp*.), dragonfly larvae including *Anax imperator mauricianusIschnura senegalensisPantala flavescensOrthetrum sp*. and *Diplacodes lefebvrii*, and tadpoles. In aquatic habitats positive for mosquito larvae the numbers of other organisms present (for example, fish and aquatic macroinvertebrates) were generally either very low or absent, thereby providing safe conditions for mosquito larvae to thrive. This finding is consistent with previous investigations, which have shown that gravid females of malaria vectors may choose adaptively between oviposition sites with and without predators [25-27]. One key determinant of the presence of *An. arabiensis* larvae may be the preference exhibited by gravid mosquitoes for oviposition with respect to some attribute(s) of the aquatic habitat [22-24]. In addition, although oviposition had probably occurred at a different time during the study period, most of the predator species we observed within different pools have much longer generation times than mosquitoes [20]. Therefore, high predation of both egg and early larval instars might also explain the apparent absence of larvae from some aquatic habitats and generally should be considered to explain patterns of larval abundance [28-32].

During the preliminary field survey reported here, larval abundance was difficult to detect reliably because of the exceedingly low number of larvae collected per dip over the survey period. It was important to further gain comprehensive understanding of whether the factors that determine the presence of larvae in potential breeding sites also affect larval abundance. Consistently in different natural and man-made larval habitats, one key point of interest was the low numbers of larvae per dip (ranging from 0 to 11 larvae) over the survey period. This is exceptionally low, in comparison with data recorded elsewhere [15-20]. The interpretation of this finding can likely be related to the environmental aquatic constraints *Anopheles arabiensis* immatures withstand in the different types of aquatic habitats examined. By contrast with studies of *Anopheles* larval ecology in African countries [20,21,33-35], application of the larval abundance index to estimate the productivity of *An. arabiensis* of different habitat types may be difficult in the context of La Reunion due to the low number of observed immatures. Within both habitat types, however, few water properties are likely to have influenced larval abundance.

One potential explanation for the low productivity observed in our study sites may be the wide use of insecticides in local agriculture as well as by vector control interventions, which have been actively implemented for many years [14]. Considering the importance of abiotic factors, the average temperatures in water bodies where the larvae were collected ranged from 29°C to 33°C, (Maximum: 39°C) in some rock pools. Larvae exposure to such high temperature could imply faster larvae development [18,36,37]. Although the relatively high temperatures we recorded may not persist throughout the day or throughout the study period [38,39], it had been reported that high temperatures of about 30°C–32°C could be harmful on a proportion of individual larvae with low thermo-tolerance [40]. However, laboratory-based studies are still necessary to precisely explain this phenomenon in *An. arabiensis*. Natural regulation mechanisms of mosquito populations in aquatic habitats such as interspecific competition can also be considered. Previous work on *Anopheles gambiae s.l.* indicated that 98% of the total mortality of larvae could be attributable to such predators, including, but not limited to Dragonflies, Backswimmers (*Notonectidae*), and predatory aquatic beetles (*Dineutus* - *Gyrinidae*) [28,41]. While this could provide more evidence for low larval abundance, our results further showed a strong correlation (*p* < 0.05), between *Anopheles* larval density and *Cypris* (*Ostracode*) and *Hydrophilus sp*. at the two study sites (Table 3). The two microorganisms are often seen in habitats with small quantity of organic matter [42,43], but the mechanisms of their association with *An. arabiensis* immatures remain unknown.

Structural complexity made up of algal cover, and grass cover affect larval population and should also be considered as important factors in *Anopheles spp*. larval ecology [20]. Therefore, another pressure that could be linked to a specific habitat is vegetation cover, the impact of which may be spatially dependent [44-46]. Unlike the habitats that were not used by *Anopheles arabiensis,* however, the positive habitats were characterized by the presence of *Commelina diffusaPaspalum scrobiculatum* and *Chloris barbata*. We lack sufficient information to explain the basis of these associations. On the other hand, *Boehmeria penduliflora* was only found in negative habitats. This plant could perhaps provide shade, one of the conditions that had been shown to adversely affect the development of *An. arabiensis* larvae [47-49].

## Conclusions

At present, the main conclusion from this study is that factors associated with the presence of immatures are complex. Man-made and naturally occurring habitats can be very different from one another in terms of habitat structure and present varying pressures and/or benefits for *Anopheles* larvae. At least in the sample of habitat we studied, periodicity in the rhythm of egg laying by gravid females could explain a variation in the time of the larval densities in those [50]. It is possible that combined effects of climatic conditions and different biotic and abiotic factors (specific to each zone, or each aquatic environment) could produce the observed low *Anopheles arabiensis* larval densities in the studied habitats. In addition, with the larviciding programme taking place in La Reunion over several years [14], vector populations on this island may not be stable and the effective size of the population, which probably has escaped larval control, is too low. This may lead to the development of convenient control strategies to minimize the occurrence of such habitats and yield significant reductions in the risk of malaria re-emergence in La Reunion.

## Competing interests

The authors declare that they have no competing interest.

## Authors’ contributions

GLC, SB and GL participated in the study design and coordination, MR carried out the field surveys and JSD and his team participated in the field surveys, GLC assembled data, performed statistical analyses and drafted the manuscript. All authors read, revised and approved the final manuscript.
